# The predictive value of sonographic images of follicular lesions – a comparison with nodules unequivocal in FNA – single centre prospective study

**DOI:** 10.1186/s12902-016-0151-5

**Published:** 2016-12-01

**Authors:** Dorota Słowińska-Klencka, Martyna Wojtaszek-Nowicka, Stanisław Sporny, Ewa Woźniak-Oseła, Bożena Popowicz, Mariusz Klencki

**Affiliations:** 1 Department of Morphometry of Endocrine Glands, Chair of Endocrinology, Medical University of Lodz, Sterlinga Str 5, Łódź, 91-425 Poland; 2 President Stanisław Wojciechowski Higher Vocational State School in Kalisz, Kalisz, Poland

**Keywords:** Thyroid, Ultrasonography, Fine needle aspiration biopsy, Follicular lesions, Cancer, FLUS, SFN

## Abstract

**Background:**

To determine the diagnostic efficacy of ultrasonographic malignancy risk features (UMRFs) in follicular lesions (FL) in a population with low risk of malignancy in FL and to compare it with a similar analysis in a group of patients with unequivocal cytology (UC): benign lesion (BL) or malignant neoplasm (MN).

**Methods:**

Presence of UMRFs (hypoechogenicity, solid echostructure, taller-than-wide shape, pathological vascularization, irregular margins, microcalcifications and macrocalcifications) and their sets were assessed in 322 FL: 202 follicular lesions of undetermined significance (FLUS) and 120 suspicious for follicular neoplasm (SFN) and 300 nodules with UC: 200 BL and 100 MN, subsequently evaluated histopathologically.

**Results:**

Cancers were confirmed in 100% nodules in MN group (89.0% of them were papillary carcinomas - PTC), in 6.4% FLUS nodules (69.2% PTC), and in 10.8% SFN nodules (30.8% PTC). In the UC group all UMRFs occurred more frequently in cancers than in benign lesions. In the FL group only calcifications were found in cancers more frequently – macro and microcalcifications together: 34.6 vs. 11.5% (*p* = 0.001) and isolated macrocalcifications: 26.0 vs. 6.8% (*p* = 0.001); the presence of those features increased the basic risk of malignancy in FL more than 2 times. The presence of at least 2 of the following URMFs: hypoechogenicity, solid echostructure, any type of calcifications and suspected shape, additionally improved sensitivity.

**Conclusions:**

Evaluation of UMRFs in FLs is less effective than in nodules with UC, and its effectiveness decreases parallel to the decrease in percentage of PTCs among malignant neoplasms and to the increase of the percentage of adenomas among benign nodules. The presence of macrocalcifications in such FLs significantly increases the basic risk of malignancy in these nodules.

## Background

Preoperative diagnostics of thyroid nodules is still a subject of investigation. Among others, the usefulness of classic ultrasonography is evaluated in the selection of nodules for fine-needle aspiration biopsy (FNA) or in assisting clinical decision making in patients with equivocal FNA results – mainly in patients with follicular lesions (FL) [1–10].

Indications for nodule biopsy are based largely on the presence of ultrasound malignancy risk features (UMRFs). These features separately do not have a satisfying sensitivity and specificity, so the use of various sets of the features is proposed. There is however some disagreement on both the significance of particular features and their optimal association. The American Thyroid Association (ATA) recommends a several grade scale for the assessment of ultrasonographic malignancy risk in nodules [11]. This scale is based mainly on the presence of high specificity UMRFs in hypoechoic nodules. These features include irregular margins, microcalcifications, taller than wide shape, disrupted rim calcifications or evidence of extrathyroidal extension. The American Society of Radiologists in Ultrasound additionally enumerates coarse calcifications among UMRFs [12], while The British Thyroid Association adds intranodular vascularity and proposes their own system to interpret various sets of UMRFs [13].

Particular discrepancies regard effectiveness of the analysis of sonographic features to estimate risk of malignancy in two categories of FL: follicular lesion of undetermined significance - FLUS –group III of cytological diagnoses in the Bethesda system and suspicious for follicular neoplasm – SFN –group IV of cytological diagnoses [1, 2, 7, 10, 14–19]. For the patients with FLUS in cytological outcome this issue is of particular importance, as this result has not been established as a direct indication for surgical treatment [11, 20]. In some reports a high positive predictive value was observed in the presence of UMRFs in FLUS nodule, but they came from the centres with high risk of malignancy in such nodules and high fraction of papillary carcinomas (PTC) among malignant tumours found in FLUS [3, 5, 14–16, 19]. Other reports do not confirm those observations [2, 6]. There is limited data on this subject, especially with respect to cytological data verified against histopathological examinations, which relate to populations with low risk of malignancy in FLUS and SFN nodules. In such populations FLUS and SFN diagnoses mainly correspond to non-neoplastic FL developing in a consequence of iodine shortage. The ratio of PTC to follicular carcinoma (FTC) is also lower in these populations [21]. These factors can influence the results of assessments of the usefulness of UMRFs and may be an important cause of the described discrepancies. Unfortunately, they are considered only in a minority of studies despite data indicating that PTC and FTC differ from each other in ultrasound imaging [22]. In our centre, where cytological diagnostics is performed in the population which had been exposed to iodine deficiency until only 20 years ago [23], the risk of malignancy in FLUS nodules, as determined with the results of histopathological examination, does not exceed 6% [24, 25]. This risk is concordant with the assumptions made when the FLUS category was created and is significantly lower than that in SFN nodules, which show a 10-20% malignancy risk [25, 26].

Thus, the aim of our study was to determine the diagnostic efficacy of UMRFs analysis in patients with FL and a relatively low risk of malignancy and to compare it with the results of similar analysis in the group of patients with unequivocal FNA outcomes: benign lesion (BL), malignant neoplasm (MN).

## Methods

### Examined patients

FNA and ultrasound imaging were performed in one centre in years 2010–2015 in patients referred by endocrinologists from outpatient clinics. Analysis of UMRFs was done prospectively. The presence of particular UMRFs was assessed by 3 experienced (>10 years) sonographers directly before FNA. Biopsy was performed only on thyroid nodules with a diameter of at least 5 mm, which had at least one malignancy risk factor (ultrasonographic or clinical), or nodules > 1 cm in the absence of more suspicious lesions. Smears were fixed in 95% ethanol solution and stained with hematoxylin and eosin. A detailed description of the FNA procedure was presented in our earlier report [24]. Results of the FNA were classified into 6 groups defined in the Bethesda system [20].

The category of biopsy result was the basis for assigning a nodule into the analysis. The study included all FLUS and SFN nodules with full ultrasound imaging data and the results of the postoperative histopathological examination. Diagnosis of SFN was formulated when the specimen contained a monotonous population of TFC arranged in three-dimensional groups and microfollicles with nuclear overlapping and crowding in a background of little to no colloid. The diagnosis of FLUS was made when the specimen showed features of both a benign thyroid nodule and a follicular neoplasm. In 4 cases specimens with limited cellularity but with nuclear atypia were also classified into category III of the Bethesda system. Additionally, a similar number was analysed in subsequent nodules with unequivocal cytology (UC): BL or MN, which were eventually subjected to surgical treatment. In the case of diagnosis of BL or FLUS surgical treatment was performed due to the large size of the nodule or based on the patient’s preference. In some cases (38, 18.8%) of FLUS nodules, surgical treatment was performed after control FNA, which gave the same diagnosis or result belonging to the category of higher risk of malignancy. Eventually the analysis was performed in 322 FL, including 202 FLUS and 120 SFN as well as in 300 UC nodules, including 200 BL and 100 MN (Table 1). Patients previously treated with surgery or radioiodine were excluded from the study.

**Table 1:** Demographic data of the patients and the percentage of cancers revealed in the nodules with unequivocal and equivocal FNA results

Parameter	Category of FNA			
	UC		FL	
	BL	MN	FLUS	SFN
Number of patients	200	100	202	120
Age - mean ± SD [years]	53.7 ± 11.4	50.3 ± 14.1	52.8 ± 13.7	52.4 ± 14.9
Number (%) of males	13 (6.5)	8 (8.0)	19 (9.4)	15 (12.5)
Volume of nodules -mean ± SD [cm^3^]	8.5 ± 13.3	2.6 ± 4.6^a^	6.4 ± 12.6	5.3 ± 11.5
% of cancers in histopathological outcome	0	100	6.4	10.8
% of PTCs in malignant neoplasms	0	89.0	69.2	30.8
% of adenomas in histopathologically benign lesions	5.5	0	11.4	25.8

*BL* benign lesion, *FL* follicular lesion, *FLUS* follicular lesions of undetermined significance, *MN* malignant neoplasm, *PTC* papillary carcinoma, *SFN* suspicious for follicular neoplasm, *UC* unequivocal cytology

^a^ *p* < 0.01 MN vs BL; the distribution of small (<1 cm) nodules between UC and FL groups was even

### Analysis of UMRFs

The US examinations were accomplished using the Aloka Prosound Alpha 7 ultrasonograph, ALOKA co. Ltd., Tokyo, Japan with a 7.5–14 MHz linear transducer. The presence of the following UMRFs was assessed: 1) hypoechogenicity or marked hypoechogenicity (compared with the surrounding thyroid or strap muscles), 2) solid echostructure (<25% cystic), 3) the cumulative presence of the features 1. and 2. 4) more-tall-than-wide shape (measured on a transverse view), 5) pathological vascularization - chaotic intranodular vascular spots, 6) suspicious margins - irregular or suggesting extrathyroidal extension, 7) calcifications with separate consideration for microcalcifications (7a) – defined as calcifications of less than 2 mm in diameter, without acoustic shadow - and macrocalcifications (defined as all other types of calcifications) without accompanying microcalcifications (7b). The occurrence of the following sets of the features was analysed:

A) >2UMRFs from the features 1–7 in 2 variants: considering microcalcifications (A1) or all calcifications (A2).
B) >2UMRFs from the features 1–7 excluding pathological vascularization in 2 variants: considering microcalcifications (B1) or all calcifications (B2). Variant B1 corresponds to category 4C (3 or 4 UMRFs) or 5 (5 UMRFs) according to the thyroid imaging reporting and data system (TIRADS) proposed by Kwak et al. [27]. These are the criteria for moderate (>50% - category 4C) and high (>90% - category 5) risk of malignancy.
C) presence of a solid hypoechoic nodule or a solid hypoechoic component in a partially cystic nodule (feature 3.) with one or more of the following features: 4, 6, 7a. That is the criterion for high risk of malignancy (>70%) adopted in the new ATA recommendations [11] with exception of disrupted rim calcifications, which were not assessed separately in our study.
D) presence >2 UMRFs from features 1–2 and those other features which would be significant in predicting malignancy of FL.

Diagnostic efficacy of particular UMRFs and their sets was analysed to differentiate between benign and malignant nodules in patients with FL and UC.

### Statistical analysis

The statistical analysis was performed with Statistica, version 10 statistical software. The comparison of frequency distributions was performed with *χ* ^2^ test (with modifications suitable for the number of analysed cases). Kruskal-Wallis test was used for comparing continuous variables between groups. Associations between US features and malignancy were evaluated by using logistic regression analysis, odds ratios (OR) with relative 95% confidence intervals (95% CI) were calculated to determine the relevance of all potential predictors of the outcome. The effectiveness of UMRFs in the differentiation between benign nodules and cancers was assessed by receiver operating characteristics curve (ROC) and the area under the ROC (AUC) value analysis. Sensitivity (SEN), specificity (SPC), positive predictive value (PPV), and negative predictive value (NPV) were analysed for established UMRFs and their sets. The percentage of nodules that satisfied evaluated criteria or their sets were determined. The value of 0.05 was assumed as the level of significance.

The study design was approved by the Local Bioethics Committee and all the patients gave their informed consent.

## Results

Table 1 shows demographic data of the examined patients and the percentage of malignant neoplasms revealed in postoperative histopathological examination in the FL and UC groups. The histopathological examination confirmed all unequivocal FNA results (BL and MN). Cytological diagnosis of MN corresponded to 89 (89.0%) PTCs (including 33/37.1% follicular variant of PTC), 9 (9.0%) medullary cancers, 1 (1.0%) FTC oxyphilic type, 1 (1.0%) undifferentiated carcinoma. In the group of FLUS nodules eventual diagnoses were: 13 cancers (6.4%) including 9 (69.2%) PTCs (5/55.5% follicular variant of PTC), 3 (23.1%) oxyphilic type of FTCs and 1 (7.7%) poorly differentiated carcinoma. In the nodules diagnosed as SFN, the postoperative examination showed 13 (10.8%) cancers including 4 (30.8%) PTCs (all follicular variant), 5 (38.5%) FTCs, 3 (23.1%) oxyphilic type of FTCs, and 1 (7.7%) undifferentiated carcinoma. In total, PTCs constituted 50.0% of all malignant neoplasms in the FL group and 89.0% of all cancers in the MN group (*p* < 0.0001). Benign neoplasms were found in 11 (5.5%) nodules with cytological diagnosis of BL – less often than in FLUS nodules - 23 (11.4%, *p* = 0.034) and in both those cytological groups less often than in SFN nodules - 31 (25.8%, *p* < 0.0001 and *p* = 0.001, respectively). In the FL group adenomas constituted 16.8% of all benign nodules (*p* < 0.0001 vs. BL).

**Fig. 1:**
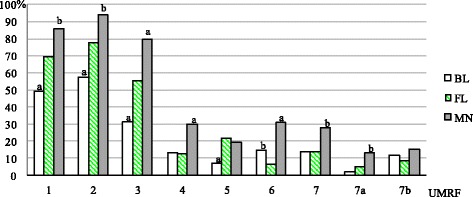
Comparison of UMRF’s incidence in ultrasound image of FL and UC (BL and MN) nodules. a – *p* < 0.0001 vs. FL. b – *p* < 0.005 vs. FL. BL - benign lesion; FL – follicular lesion; MN - malignant neoplasm; UMRF: 1 - hypoechogenicity, 2 - solid echostructure, 3 - the features 1. and 2., 4 - taller-than-wide shape, 5 - pathological vascularization, 6 - suspicious margins, 7 - calcifications, 7a – microcalcifications, 7b - macrocalcifications without accompanying microcalcifications

Figure 1 shows differences in ultrasound image between FL and nodules with cytological diagnoses of BL and MN. FL nodules were less often than BL, and more often than MN, hypoechoic: 69.5 vs. 49.0% and 86.0% (*p* < 0.0001 and *p* < 0.001, FL vs. BL and MN, respectively), solid: 78.0 vs. 57.5% and 78.0% (*p* < 0.0001 and *p* < 0.001, FL vs. BL and MN, respectively), both solid and hypoechoic: 55.3 vs. 31.5% and 80.0% (*p* < 0.0001 in both cases, FL vs. BL and MN, respectively). Also, FL were less often taller than wide than MN: 12.7 vs. 30.0% (*p* < 0.0001), and less frequently contained any type of calcifications: 13.4 vs. 28.0% (*p* < 0.005) or microcalcifications: 5.0 vs. 13.0% (*p* < 0.005). Suspected vascularization occurred more often in FL than in BL: 21.7 vs. 7.0% (*p* < 0.0001). Irregular margins were observed in FL occasionally, significantly less often than in BL and MN: 6.2 vs 14.5% and 31.0% (*p* < 0.005 and *p* < 0.0001, respectively).

Table 2 shows the incidence of particular UMRFs in UC and FL nodules in relation to the histopathological outcome: thyroid cancer vs. benign lesion. In the UC group all UMRFs (features 1–7) occurred more often in cancers than in benign nodules with the exception of isolated macrocalcifications. In the FL group the only feature more common in cancers than in benign nodules was the presence of any type of calcifications: 34.6 vs. 11.5% (*p* = 0.001) as well as isolated macrocalcifications: 26.0 vs. 6.8% (*p* = 0.001), with no difference for microcalcifications. Noteworthy, taller than wide shape was the closest to the threshold of significance of difference between benign and malignant nodules in the FL group: 23.1 vs. 11.8% (*p* = 0.098). In FLUS nodules (Table 3) UMRFs other than calcification and suspected shape occurred slightly more often in cancers than in benign nodules (the smallest differences were observed for irregular margins). In SFN nodules hypoechogenicity and suspected margins were observed slightly more often in benign nodules, while hypoechogenicity of solid nodule, pathological vascularization and microcalcifications were equally frequent in benign and malignant nodules.

**Table 2:** Comparison of the incidence of UMRFs and their sets in the nodules histopathologically benign and malignant in UC and FL groups

Sonographic feature	Category of FNA					
	UC (300)			FL (322)		
	Histopathological results			Histopathological results		
	Benign (200) No/%	Malignant (100) No/%	*p*	Benign (296) No/%	Malignant (26) No/%	*p*
1 –hypoechogenicity	98/49.0	86/86.0	**<0.0001**	204/68.9	19/73.1	0.659
2 - solid echostructure	115/57.5	94/94.0	**<0.0001**	227/76.7	24/92.3	0.111
3 (1 & 2) - hypoechogenicity of solid nodule	63/31.5	80/80.0	**<0.0001**	161/54.4	17/65.4	0.279
4 - taller-than-wide shape	26/13.0	30/30.0	**<0.0001**	35/11.8	6/23.1	0.098
5 - pathological vascularization	14/7.0	19/19.0	**<0.0001**	63/21.3	7/26.9	0.504
6 - suspicious margins	29/14.5	31/31.0	**<0.0001**	19/6.4	1/3.8	0.922
7 – calcifications (micro or macro)	27/13.5	28/28.0	**<0.0001**	34/11.5	9/34.6	**0.001**
7a – microcalcifications	4/2.0	13/13.0	**<0.0001**	14/4.7	2/7.7	0.845
7b – isolated macrocalcifications	23/11.5	15/15.0	0.535	20/6.8	7/26.0	**0.001**
Sets of sonographic features						
A1 - >2 UMRFs of: 1, 2, 4, 5, 6, 7a	30/15.0	55/55.0	**<0.0001**	71/24.0	8/30.8	0.441
A2 - >2 UMRFs of: 1, 2, 4, 5, 6, 7	40/20.0	63/63.0	**<0.0001**	79/26.7	12/46.2	**0.035**
B1 - >2 UMRFs of: 1, 2, 4, 6, 7a (TIRADS)	25/12.5	46/46.0	**<0.0001**	37/12.5	6/23.1	0.128
B2 - >2 UMRFs of: 1, 2, 4, 6, 7	33/16.5	53/53.0	**<0.0001**	46/15.5	10/38.5	**0.003**
C - 3 with 4 or 6 or 7a (ATA)	26/13.0	46/46.0	**<0.0001**	36/12.2	5/19.2	0.299
D - >2UMRFs of: 1, 2, 4, 7	20/10.0	43/43	**<0.0001**	37/12.5	10/38.5	**0.001**

Statistical significance was marked with bold type

*FL* follicular lesion, *UC* unequivocal cytology, *UMRFs* ultrasonographic malignancy risk features

**Table 3:** Comparison of the incidence of UMRFs and their sets in the nodules histopathologically benign and malignant in FLUS and SFN groups

Sonographic feature	Category of FNA					
	FLUS (202)			SFN (120)		
	Histopathological results			Histopathological results		
	Benign (189) No/%	Malignant (13) No/%	*p*	Benign (107) No/%	Malignant (13) No/%	*p*
1 – hypoechogenicity	127/67.2	11/84.6	0.318	77/72.0	8/61.5	0.435
2 - solid echostructure	137/72.5	11/84.6	0.527	90/84.1	13/100.0	0.258
3 (1 & 2) -hypoechogenicity of solid nodule	94/49.7	9/69.2	0.299	67/62.6	8/61.5	0.939
4 - taller-than-wide shape	18/9.5	3/23.1	0.281	17/15.9	3/23.1	0.793
5 - pathological vascularization	37/19.6	4/30.8	0.539	26/24.3	3/23.1	0.806
6 - suspicious margins	13/6.9	1/7.7	0.651	6/5.6	0/0.0	0.839
7 – calcifications (micro or macro)	17/9.0	4/30.8	**0.044**	17/15.9	5/38.5	**0.047**
7a – microcalcifications	7/3.7	1/7.7	0.983	7/6.5	1/7.7	0.666
7b – isolated macrocalcifications	10/5.3	3/23.1	0.052	10/9.3	4/30.8	0.069

Statistical significance was marked with bold type

*FL* follicular lesion, *UC* unequivocal cytology, *UMRFs* ultrasonographic malignancy risk features

Logistic regression analysis confirmed that the presence of isolated macrocalcifications and the presence of any type of calcifications were the only independent UMRFs in differentiating between benign and malignant FL nodules (Table 4). In the UC group the independent features suggesting malignancy were hypoechogenicity, solid echostructure, taller-than-wide shape, pathological vascularization, and microcalcifications. Univariate analysis also showed the significance of suspected margins and any type of calcifications, and demonstrated no significance of isolated macrocalcifications.

**Table 4:** Results of logistic regression analysis in UC and FL nodules for particular UMRFs

Sonographic feature	Category of FNA			
	UC OR (95% Cl) *p*		FL OR (95% Cl) *p*	
	univariate analysis	multivariate analysis	univariate analysis	multivariate analysis
1 – hypoechogenicity	6.5 (3.2–13.1) **<0.0001**	4.4 (2.1–9.3) **<0.0001**	3.1 (0.7–13.5) 0.720	1.0 (0.4–2.8) 0.948
2 - solid echostructure	11.1 (4.3–28.7) **<0.0001**	10.1 (3.6–28.3) **<0.0001**	1.2 (0.5–3.2) 0.140	3.9 (0.8–18.4) 0.082
3 (1 & 2) - hypoechogenicity of solid nodule*	8.7 (4.6–16.3) **<0.0001**	7.0 (3.6–13.5) **<0.0001**	1.5 (0.6–3.6) 0.400	1.5 (0.6–3.9) 0.363
4 - taller-than-wide shape	2.9 (1.5–5.4) **0.001**	2.3 (1.1–4.9) **0.022**	2.2 (0.8–6.3) 0.149	2.0 (0.6–6.0) 0.404
5 - pathological vascularization	3.1 (1.4–6.7) **0.005**	3.4 (1.3–8.4) **0.009**	1.4 (0.5–3.6) 0.536	1.5 (0.5–4.6) 0.382
6 - suspicious margins	2.7 (1.4–5.0) **0.002**	1.2 (0.4–2.4) 0.663	0.7 (0.1–5.4) 0.722	0.3 (0.1–2.4) 0.263
7 – calcifications (micro and macro)**	2.4 (1.3–4.6) **0.006**	1.7 (0.8–3.5) 0.160	4.4 (1.7–11.3) **0.002**	4.8 (1.8–12.9) **0.002**
7a-microcalcifications	7.0 (2.1–23.1) **0.001**	5.0 (1.2–20.7) **0.024**	2.0 (0.4–9.7) 0.369	3.2 (0.6–17.4) 0.167
7b – isolated macrocalcifications	1.4 (0.6–2.9) 0.425	1.1 (0.5–2.6) 0.855	5.1 (1.8–14.6) **0.002**	7.4 (2.3–23.7) **0.001**

Statistical significance was marked with bold type

*FL* follicular lesion, *UC* unequivocal cytology

*multivariate analysis did not consider features 1 and 2 separately

**multivariate analysis did not consider features 7a and 7b separately

Analysis of diagnostic efficacy of UMRFs (Table 5) showed that in both groups solid echostructure and hypoechogenicity had high SEN (UC: 94.0 and 86.0%, FL: 92.3 and 73.1%, respectively) and low SPC (UC: 42.5 and 51.0%, FL: 23.3 and 31.1%, respectively). More than 60% of nodules in both groups presented with those features. Other single features were found in at most 20% of UC nodules and 21.7% of FL nodules. They had at least 85.5% SPC in the UC group and at least 78.7% SPC in the FL group, while SEN was low (13.0–31.0% in UC group and 3.8–34.6% in FL group). The lowest SEN was observed for microcalcifications in the UC group and for irregular margins in the FL group. The PPV reached the highest values for microcalcifications in the UC group (76.5%), and for macrocalcifications in the FL group (25.9%) (Table 5). The evaluation of single UMRFs was characterized in both groups by low or medium accuracy – AUC in the range 0.5–0.7. In the FL group the highest AUC was observed for the presence of any type of calcifications: 0.624 (0.489–0.759, CI95%, *p* = 0.071), and in the UC group for the presence of solid, hypoechoic nodules: 0.749 (0.686–0.811, CI95%, *p* < 0.0001).

**Table 5:** Values of indexes describing the diagnostic efficacy of particular UMRFs and their sets in UC and FL nodules

Sonographic feature	Category of FNA									
	UC					FL				
	SEN [%]	SPC [%]	PPV [%]	NPV [%]	% of nodules	SEN [%]	SPC [%]	PPV [%]	NPV [%]	% of nodules
1 – hypoechogenicity	86.0	51.0	46.7	87.9	61.3	73.1	31.1	8.5	94.8	69.3
2 - solid echostructure	94.0	42.5	45.0	93.4	69.7	92.3	23.3	9.6	97.2	77.9
3 – (1 & 2)- hypoechogenicity of solid nodule	80.0	68.5	55.9	87.3	47.7	65.4	45.6	9.5	93.8	55.3
4 - taller-than-wide shape	30.0	86.5	53.6	71.3	18.7	23.1	88.2	14.6	97.8	12.7
5 - pathological vascularization	19.0	93.0	57.6	69.7	11.0	26.9	78.7	10.0	92.5	21.7
6 - suspicious margin	31.0	85.5	51.7	71.3	20.0	3.8	93.6	5.0	91.7	6.2
7 – calcifications (micro or macro)	28.0	86.5	50.9	70.6	18.3	34.6	88.5	20.9	93.9	13.4
7a – microcalcifications	13.0	98.0	76.5	69.3	5.7	7.7	95.3	12.5	98.7	5.0
7b – isolated macrocalcifications	15.0	88.5	39.5	67.6	12.7	26.9	93.2	25.9	93.6	8.4
Sets of sonographic features										
A1 - >2 UMRFs of: 1, 2, 4, 5, 6, 7a	55.0	85.0	64.7	79.1	28.3	30.8	76.0	10.1	92.6	24.5
A2 - >2 UMRFs of: 1, 2, 4, 5, 6, 7	63.0	80.0	61.1	81.2	34.3	46.2	73.3	13.2	93.9	28.3
B1 - >2 UMRFs of: 1, 2, 4, 6, 7a (TIRADS)	46.0	87.5	64.8	76.4	23.7	23.1	87.5	14.0	92.8	13.4
B2 - >2 UMRFs of: 1, 2, 4, 6, 7	53.0	83.5	61.6	78.0	28.7	38.5	84.5	17.9	94.0	17.4
C - 3 with 4 or 6 or 7a (ATA)	46.0	87.0	63.9	76.3	24.0	19.2	87.8	12.2	92.5	12.7
D - >2 UMRFs of: 1, 2, 4, 7	43.0	90.0	66.2	75.9	21.0	38.5	87.5	21.3	94.2	14.6

*FL* follicular lesion, *UC* unequivocal cytology, *UMRFs* ultrasonographic malignancy risk features

Tables 2 and 5 show data on the incidence and diagnostic efficacy of UMRFs sets in both groups. In the FL group the highest sum of SEN and SPC (38.5 and 87.5%, respectively) and the highest PPV (21.3%) were found for the set D composed of 2 features with high SEN: hypoechogenicity and solid echostructure and 2 features with high SPC: any type of calcifications and taller-than-wide shape. The addition of suspected margins to that set did not improve SEN (38.5%), and slightly lowered SPC (84.5%) and PPV (17.9%). Further insertion of suspected vascularization into the set increased SEN to 46.2%, but lowered SPC to 73.3%, and PPV to 13.2%. The incidence of the sets of features including microcalcifications did not differ significantly between benign and malignant nodules in the FL group. The set proposed by the ATA (C) and the TIRADS set (B1) showed SEN below 25% in the FL group, with SPC similar to that of the set D. In the UC group, all the analysed sets of UMRFs occurred more often in cancers than in benign nodules (*p* < 0.0001 in all cases). Analysis of the sets of UMRFs in the UC group allowed for approx. 1.5–5 time increase in SEN in comparison with the evaluation of single features of low SEN, while keeping SPC in the range of 80.0–90.0%. PPV exceeded 60% in the case of all evaluated sets of features.

Table 6 shows the differences in ultrasound image of malignant neoplasms from the UC and FL groups, as well as benign nodules in those groups. Both cancers and benign nodules in the FL group had suspected margins less often than their counterparts from UC group – cancers: 3.8 vs. 31.0% (*p* = 0.010), benign lesions: 6.4 vs. 14.5% (*p* = 0.003). Cancers in SFN subgroup were less often hypoechoic than cancers in the MN subgroup: 61.5 vs. 86.0% (*p* = 0.026). Cancers of the FL group showed presence of all examined sets of UMRFs, which contained microcalcifications (the sets A1, B, C, *p* < 0.05 in all cases) less often than cancers of the UC group. Benign nodules of the FL group were hypoechoic: 68.9 vs. 49.0%, solid: 76.7 vs. 57.5%, both solid and hypoechoic: 54.4 vs. 31.5% and pathologically vascularized: 21.3 vs. 7.0% more often than benign nodules of UC group (*p* < 0.0001 in all cases). Those differences were significant also when benign nodules of the FLUS and SFN subgroups were considered separately and compared to the UC group (*p* < 0.005 in all cases). No significant differences were observed between cancers of the FLUS and SFN subgroups. Benign nodules in the SFN subgroup were more often solid and hypoechoic than benign nodules in the FLUS subgroup: 62.6 vs. 49.7% (*p* = 0.033).

**Table 6:** Comparison of the incidence of UMRFs and their sets in malignant neoplasms and benign nodules in relation to the FNA result – UC vs. FL

Sonographic feature	Results of histopathological examinations					
	Benign			Malignant		
	Category of FNA			Category of FNA		
	UC (200) No/%	FL (296) No/%	*p*	UC (100) No/%	FL (26) No/%	*p*
1 –hypoechogenicity	98/49.0	204/68.9	**<0.0001**	86/86.0	19/73.1	0.115
2 - solid echostructure (<25% cystic)	115/57.5	227/76.7	**<0.0001**	94/94.0	24/92.3	0.892
3 (1 & 2) -hypoechogenicity of solid nodule	63/31.5	161/54.4	**<0.0001**	80/80.0	17/65.4	0.115
4 -taller-than-wide shape	26/13.0	35/11.8	0.696	30/30.0	6/23.1	0.486
5 -pathological vascularization	14/7.0	63/21.3	**<0.0001**	19/19.0	7/26.9	0.374
6 - suspicious margins	29/14.5	19/6.4	**0.003**	31/31.0	1/3.8	**0.010**
7 –calcifications (micro or macro)	27/13.5	34/11.5	0.503	28/28.0	9/34.6	0.509
7a –microcalcifications	4/2.0	14/4.7	0.177	13/13.0	2/7.7	0.686
7b – isolated macrocalcifications	23/11.5	20/6.8	0.065	15/15.0	7/26.0	0.154
Sets of sonographic features						
A1 - >2 UMRFs of: 1, 2, 4, 5, 6, 7a	30/15.0	71/24.0	**0.015**	55/55.0	8/30.8	**0.028**
A2 - >2 UMRFs of: 1, 2, 4, 5, 6, 7	40/20.0	79/26.7	0.087	63/63.0	12/46.2	0.119
B1 - >2 UMRFs of: 1, 2, 4, 6, 7a (TIRADS)	25/12.5	37/12.5	1.0	46/46.0	6/23.1	**0.034**
B2 - >2 UMRFs of: 1, 2, 4, 6, 7	33/16.5	46/15.5	0.775	53/53.0	10/38.5	0.187
C - 3 with 4 or 6 or 7a (ATA)	26/13.0	36/12.2	0.782	46/46.0	5/19.2	**0.013**
D - >2 UMRFs of: 1, 2, 4, 7	20/10.0	37/12.5	0.392	43/43.0	10/38.5	0.676

Statistical significance was marked with bold type

*FL* follicular lesion, *UC* unequivocal cytology, *UMRFs* ultrasonographic malignancy risk features

## Discussion

The aim of the study was the analysis of efficacy of UMRFs evaluation in patients with FL. Data published on this issue is less concordant than that referring to the usefulness of UMRFs analysis in the whole group of thyroid nodules. However FL nodules are special, and in routine cytological examinations there is no possibility to differentiate between malignant and benign nodules, especially between FTC and adenoma [11, 20]. Such a group of nodules raises expectations from ultrasound imaging. There is a continuous search for features that could aid in making clinical decisions and avoiding unnecessary surgical treatment. This is of particular importance in populations similar to our own, characterized with a low risk of malignancy in FL nodules and relatively high percentage of FTCs among malignant tumours. In our study, FTCs amounted to 46.2% of malignant neoplasms in the FL group. The percentage of PTCs among malignant nodules in FL group was nearly 40% lower than in the group of unequivocal diagnosis of MN. Among histopathologically benign nodules, the incidence of follicular adenomas was 3 times higher in the FL group than in the nodules with unequivocal cytological diagnosis of BL.

Unfortunately, our data indicate that the evaluation of UMRFs in such FLs is less effective, and its effectiveness decreases parallel to the decrease in percentage of PTCs among malignant neoplasms and to the increase of the percentage of adenomas among benign nodules. In the group of UC nodules all the examined UMRFs were found more often in cancers than in benign nodules. Similar results were shown in the metaanalysis by Remonti et al. [8]. On the other hand, in the group of FL the features observed more often in cancers than in benign lesions included only calcifications of any type and macrocalcifications without accompanying microcalcifications. In the FLUS subgroup, in which PTCs constituted about 70% of all malignant neoplasms, other UMRFs also occurred slightly more often in cancers. In contrast, in the SFN subgroup where the percentage of PTC only slightly exceeded 30% and adenomas constituted above 25% of benign nodules, the relation was reversed: hypoechogenicity, suspected margins, and - to a lesser degree - hypoechogenicity of solid nodule and pathological vascularisation were observed slightly more often in benign nodules.

Our study suggests that the differences in the effectiveness of UMRFs assessment between the FL and UC groups are the consequence of different ultrasound image of both cancers and benign nodules between these groups. Cancers in the FL group had suspected margins less often than cancers in the UC group. Additionally, cancers in the SFN subgroup were less frequently hypoechoic than cancers in the UC group. On the other hand, benign nodules diagnosed cytologically as FLUS or SFN were more often hypoechoic, solid as well as pathologically vascularized, and had suspected margins less often than their counterparts with an unequivocal BL diagnosis. Other authors also reported the differences between ultrasound images of FTCs and PTCs. Jeh et al. [22] showed that FTCs usually had regular margins, were less frequently hypoechoic and solid than PTCs, less often presented suspicious shape or margins, and were not characterized by microcalcifications. The follicular variant of PTC, which more often corresponded to malignant nodules in FL group than to those in MN group, causes diagnostic difficulties, as it shows a higher rate of follicular-like features than the conventional variant [28, 29]. Thus, the obtained results cannot be surprising if one considers the fact that UMRFs have been established mainly on the basis of ultrasound image of the most common PTCs and are optimized for revealing that type of thyroid cancer [27, 30].

Many reports on the usefulness of the UMRFs assessment, both in unselected thyroid nodules and in FL nodules, and particularly in nodules of the category III in the Bethesda system, come from the countries with very high iodine supply (e.g. South Korea). In such areas PTCs dominate not only in the group of nodules with unequivocal cytological diagnosis of MN, but also among FL nodules, and especially in nodules of the category III in the Bethesda system [6, 14–16, 19]. The percentage of PTCs in this category is further increased by frequent classification into this category of the nodules with borderline cytological result, when characteristic features of benign and malignant lesions coexist in a smear and the cellularity of the aspirate is scant. Consequently, at some centres the frequency of formulating FNA diagnoses of the category III reaches up to 20% (instead of assumed 5–7%). Additionally, the malignancy risk related to this category approaches 50% (instead of assumed 5–15%) [31–35], and the percentage of PTCs among malignant neoplasms in this category is over 90% [6, 14–16, 19]. In such centres, the effectiveness of the UMRFs assessment in the category III of FNA results is high. In the study by Jeong et al. [14] diagnostic usefulness of evaluating taller-than-wide shape, ill-defined margins, and microcalcifications or macrocalcifications was shown in the Bethesda category III nodules. Yoo et al. [19] showed that malignancy in the nodules of that category was associated with taller-than-wide shape, ill-defined margins and marked hypoechogenicity, while Gweon et al. [15] reported that it was related to marked hypoechogenicity, microlobulated or irregular margins, microcalcifications, and taller-than-wide shape. Kim et al. [16] showed that the presence of several (>1) UMRFs of the following: marked hypoechogenicity, a spiculated margin, microcalcifications, and a taller-than-wide shape in solid thyroid Bethesda III nodules, is an indication for surgical treatment without a control FNA. In all these studies, the risk of malignancy in the nodules with the category III in the Bethesda system and suspicious ultrasound image was significantly higher when compared with cytological evaluation alone. But in none of the above mentioned studies the risk of malignancy in Bethesda III nodules was assessed with consideration for whether the examined nodules were on the borderline between benign lesions and follicular neoplasms (nodules defined as classic FLUS with atypia of cellular architecture) or on the borderline between benign nodules and cancers (nodules with nuclear atypia – atypia of undetermined significance (AUS). Many authors use this distinction and indicate that the AUS subgroup is characterized by a higher than FLUS risk of malignancy, a higher percentage of PTCs among malignant neoplasms [9, 36–39] and a specific ultrasound image. Lee et al. [4] found that the AUS group more frequently had non-circumscribed margins and taller-than-wide shape than the FLUS group. The incidence of FTCs was significantly higher in the FLUS group than in the AUS group (33.3 vs.1.6%) [4]. Similarly, Choi et al. [38] found that a spiculated margin, marked hypoechogenicity, and micro- or macrocalcifications were significantly more common in AUS than in FLUS. Interestingly, in the report from Turkey, where the risk of malignancy in Bethesda III thyroid nodules is lower (22.8%) than in the above mentioned reports from South Korea, the only predictive features of malignancy were hypoechogenicity in the AUS group and peripheral vascularization in the FLUS group [2]. In the report from Brazil, with a similar risk of malignancy in Bethesda III thyroid nodules (22.6%), Rosario [9] showed that AUS presented a higher frequency of suspicious malignant US findings compared to FLUS, but evaluation of UMRFs allowed to predict malignancy both in AUS and FLUS nodules. However, in that study PTCs also constituted 91.2% of all malignant tumours.

In our study the nodules of the Bethesda category III were dominated by FLUS diagnoses. Only in 2.1% of cases the smear was classified into this category because of the presence of nuclear atypia – corresponding to AUS. It can be explained by epidemiological circumstances and a continued high incidence of non-neoplastic thyroid nodules in our patients. But it may also be the consequence of a more conservative attitude to the rules for formulation of FLUS diagnosis, which was limited to nodules from the boundary between follicular neoplasms and benign lesions. Consequently, the percentage of PTCs among malignant neoplasms was lower in our study (70%) and it could decrease the effectiveness of UMRFs evaluation.

In the case of the category IV of cytological diagnoses – SFN – the effectiveness of UMRFs analysis was even lower, as mentioned before. Recent reports seldom refer to this particular group of cytological diagnoses. This is the consequence of clinical recommendations that imply surgical treatment in such cases. Moreover, many previous reports showed that the evaluation of UMRFs was not useful in that category of cytological diagnoses [17]. Recently, Iskandar et al. [18] analysed joint groups III and IV of cytological diagnoses and found that in such a group of nodules the examined UMRFs (microcalcifications, irregular borders, hypervascularity and hypoechogenicity) were not associated with malignancy. The malignancy rate in resected thyroid nodules was 13% for Bethesda III and 28% for Bethesda IV, PTCs constituted 72% of malignant neoplasms. Park et al. [7] found that in the category IV the evaluation of UMRFs had the lowest efficiency in the comparison with other groups of cytological diagnoses (the risk of malignancy in that group was 5.7%). On the other hand, in the group of nodules of the IV diagnostic category and with 24.3% overall malignancy rate Chng et al. [1] showed the usefulness of assessing irregular margins, hypoechogenicity, and taller-than-wide shape, despite the fact that the percentage of PTCs among malignant neoplasms was below 50% in that study. However, a noticeable amount (40%) of the patients in that group were not treated surgically.

In our study only the presence of macrocalcifications or any type of calcifications (which obviously included macrocalcifications) increased the risk of malignancy in FL nodules (both FLUS and SFN) in comparison with cytological evaluation alone to the values >15% (PPV for any type of calcifications - 25.9%, and for macrocalcifications - 20.9%), which is a threshold commonly assumed in the recommendations above which the surgical treatment is advocated. Only slightly lower PPV (14.6%) was found for taller-than-wide shape. Similar PPV values were obtained for the set of UMRFs including 2 features with high SEN: hypoechogenicity, solid echostructure and 2 features with high SPC: calcifications of any type and suspected shape. That set showed nearly 90% SPC and SEN higher than the assessment of calcifications alone. Higher SEN was also observed in all the examined sets which included calcifications of any type. The sets of features which included only microcalcifications were less effective in the FL group, and the incidence of such sets was similar in benign and malignant nodules in that group. The lowest values were observed for the set proposed by the ATA and the TIRADS set. But both those sets are tailored to reveal the most common PTCs. The TIRADS set was specified by Kwak in the group of nodules with unequivocal cytological result after excluding nodules with indeterminate cytology [27] and verified against the group of cancers with a low percentage of FTCs (1.7%) and low percentage of follicular variant of PTCs (2.5%) [40]. Papillary cancers amounted to more than 95% of malignant neoplasms in that study. The authors found in such a group of thyroid nodules that the presence of macrocalcifications without accompanying microcalcifications was not useful diagnostically [27], what is in concordance with our observations in the group of nodules with unequivocal cytology.

Recently, several papers have been published on the evaluation of TIRADS in FL nodules. Yoon et al. [10] found significant differences in TIRADS category between benign and malignant nodules in the AUS subgroup, but not in the FLUS subgroup. In the group of nodules with FLUS in cytological result PTCs amounted to 57% of malignant neoplasms while in the AUS group - 100%. Park et al. [6] analysed the usefulness of TIRADS in nodules with two AUS/FLUS results and found that ultrasound features and TIRADS categories did not differ between benign and malignant nodules. Maia et al. [5] evaluated the usefulness of the TIRADS system in Bethesda categories III, IV and V with positive conclusions, but they used the modified version of the system with the addition of vascularity criteria by Doppler analysis. Also in that study, PTCs constituted nearly 90% of all malignant neoplasms. On the other hand, Chng et al. [1] showed the usefulness of TIRADS scores 4C and 5 in predicting malignancy of category IV nodules, but that study had retrospective design and nearly half of the patients did not have histological results.

In the UC group the analysis of all the sets of UMRFs increased SEN as much as several times in comparison with the evaluation of single features with low SEN, while preserving high SPC. PPV exceeded 60% for all the examined sets of features. But direct comparison of PPV between the UC and FL groups, as well as between our study and the reports from other centres is not possible. PPV of UMRFs depends on the malignancy rate of nodules in a particular diagnostic category. Thus special attention should be paid while interpreting PPV in the case of cytological category III, where the risk of malignancy ranges widely from 5 to 50% [31–35].

Other factors, which make comparison of the reported results difficult, are independent of the examined population, but are related to the expertise of the person performing the examination and the type of ultrasonograph used. Qualitative and not quantitative character of UMRFs makes them more susceptible for variable interpretation by ultrasonographers, especially when they come from different centres and work with different equipment. Another issue is the variable way of defining analysed features, e.g. solid character of a nodule is assumed when the solid part of a nodule is greater than 50% [4] or 90% [40]. In some ultrasonographs additional software is used that facilitates visualisation of some UMRFs, e.g. microcalcifications characteristic of PTCs. It should be stressed that the assessment of microcalcifications is not an easy task. Bright reflections on ultrasound imaging in spongiform nodules may be confused with microcalcifications by less proficient sonographers [11]. Thus, in our study all examinations were performed by experienced ultrasongraphers with the same equipment.

Another important advantage of our study is performing UMRFs evaluation directly prior to biopsy. Therefore, the result of FNA did not influence that evaluation. We also limited our analysis to nodules verified with postoperative histopathological examination. Such a design has both advantages (certainty of the correct diagnosis of benign and malignant lesions) and disadvantages (a bias introduced by the additional selection of nodules). But in our material the difference in the risk of malignancy in FLUS nodules, as determined by histopathological examination and cytological follow-up, is not big (<3%) [25]. Our previous study also showed that there were no differences in the ultrasound image of FLUS nodules in the patients treated surgically and conservatively. Patients treated surgically were younger and had larger nodules [25]. A disadvantage of our study is the relatively low number of cancers in the FL group, but it reflects the frequency of cancers in this cytological category in our population. Undoubtedly, extension of the study for a larger group of patients would be indicated. From the methodological point of view the ideal study design would compare the effectiveness of URMF analysis between iodine sufficient and iodine deficient populations. However such an assessment would meet practical difficulties in securing uniform URMF assessment in two geographically remote study groups.

## Conclusions

Summing up, in FL nodules with low risk of malignancy and high percentage of FTCs among malignant neoplasms the evaluation of single UMRFs, as well as their sets, shows lower efficiency than in the group of nodules with unequivocal diagnosis of BL or MN. However, the presence of macrocalcifications in the nodule justifies surgical treatment. The effectiveness of UMRFs evaluation in FL decreases parallel to the decrease in percentage of PTCs among malignant neoplasms and to the increase of the percentage of adenomas among benign nodules. The ultrasound image of follicular neoplasms differs from the image of PTCs. Thus, comparisons of the results obtained in various centres on the discussed issue are not justified without consideration for epidemiological differences between examined populations and for the differences in the way of classification of the aspirates into diagnostic categories corresponding to FL.

## Abbreviations

95% CI: 95% confidence intervals
AUC: Area under the receiver operating characteristics curve
BL: Benign lesion
FL: Follicular lesions
FLUS: Follicular lesions of undetermined significance
FNA: Fine-needle aspiration biopsy
FTC: Follicular thyroid carcinoma
MN: Malignant neoplasm
NPV: Negative predictive value
OR: Odds ratios
PPV: Positive predictive value
PTC: Papillary thyroid carcinoma
ROC: Receiver operating characteristics curve
SD: Standard deviation
SEN: Sensitivity
SFN: Suspicious for follicular neoplasm
SPC: Specificity
TIRADS: Thyroid imaging reporting and data system
UC: Unequivocal cytology
UMRFs: Ultrasonographic malignancy risk features
